# Protein–Polyphenol Interactions in Specialty Oilseeds: Multiscale Mechanisms, Physicochemical Reshaping, and Advanced Food Applications

**DOI:** 10.3390/foods15111939

**Published:** 2026-06-01

**Authors:** Yujie Mu, Nanjie Jiang, Yongrou Fang, Xiang Liu, Xia Xiang, Can Cui

**Affiliations:** 1 School of Chemical Engineering, Guizhou Minzu University, Guiyang 550025, China; 18875192825@163.com (Y.M.); m17785023430@163.com (Y.F.); 2 Oil Crops Research Institute of Chinese Academy of Agricultural Sciences, Wuhan 430062, China; jiangnj.bio@gmail.com (N.J.); 13545114344@163.com (X.L.)

**Keywords:** specialty oilseeds, protein–polyphenol interactions, multiscale characterization, functional properties, processing window, food applications

## Abstract

Specialty oilseeds, encompassing herbaceous (sunflower, flaxseed, sesame) and woody (Camellia oleifera, walnut, olive) species, serve as important sustainable sources of plant proteins that are inherently enriched with structurally diverse endogenous polyphenols such as chlorogenic acid, lignans, catechins, and ellagitannins. During processing, these polyphenols drive covalent or non-covalent interactions that profoundly reshape the physicochemical and functional properties of the resulting food systems. While prior reviews have largely remained descriptive or focused on single commodities or model proteins, this work provides the first critical, multiscale synthesis across herbaceous and woody oilseeds. We systematically compare polyphenol diversity, delineate the continuum from reversible non-covalent association (specific residue-level vs. non-specific surface-mediated) to irreversible covalent coupling, and establish a “structure–interaction–function” framework that explicitly defines a condition-dependent “Processing Window”. Within this window, moderate interactions enhance interfacial viscoelasticity, oxidative stability, foaming, and emulsification; excessive cross-linking, however, impairs solubility, digestibility, and sensory quality. By integrating experimental spectroscopy (UV-vis, FT-IR, CD, ITC), microscopic imaging, and computational simulations (molecular docking and dynamics), we map residue-level binding modes directly to macroscopic functional outcomes. Furthermore, the review evaluates the engineering potential of these complex systems in frontier applications such as antioxidant emulsions and active packaging. By explicitly identifying evidence boundaries and quantitative knowledge gaps in endogenous matrices, this work provides a comprehensive theoretical framework for the precision design and valorization of specialty oilseed-derived functional ingredients.

## 1. Introduction

In the global pursuit of sustainable plant-based nutrition, research has moved beyond conventional protein sources such as soy and pea to explore specialty oilseeds, including both herbaceous species (sunflower, flaxseed, sesame) and woody species (Camellia oleifera, walnut, olive). While bulk oilseeds dominate caloric supply, specialty oilseeds offer distinct added value through their exceptionally high concentrations of structurally diverse endogenous polyphenols—ranging from simple phenolic acids (e.g., chlorogenic acid) to complex tannins (e.g., ellagitannins and lignans) [1,2,3,4,5,6,7,8]. This chemical richness, however, presents a double-edged challenge: protein–polyphenol interactions during processing can either enhance functional properties or induce severe quality defects such as discoloration, aggregation, reduced solubility, and impaired digestibility. Much of the existing literature remains at the level of textbook descriptions derived from simplified model systems or single commodities and lacks a critical, comparative synthesis focused on the complex endogenous matrices of specialty oilseeds. There is therefore an urgent need for a multiscale framework that maps molecular binding modes directly to macroscopic food performance, thereby guiding the precision design and valorization of next-generation functional ingredients from specialty oilseed byproducts.

To ensure scientific rigor, this review adopts a precise definition of protein–polyphenol “interactions”: the direct molecular-level associations between polyphenols and the polypeptide chains of proteins. We explicitly distinguish non-specific surface adsorption (multidentate bridging or hydration-layer effects without strict residue selectivity) from residue-specific binding (high-affinity docking at defined amino acid sites). At the mesoscopic and microscopic scales, these interactions form a continuum: from reversible non-covalent associations—driven by hydrogen bonding, hydrophobic pocketing, and π–π stacking with residues such as tyrosine—to irreversible covalent coupling. The latter typically proceeds via Michael addition or Schiff-base formation between quinone intermediates (generated by polyphenol oxidation) and nucleophilic side chains such as lysine (–NH_2_) or cysteine (–SH) [3,8,9,10]. By establishing this residue-level terminology early, the review provides a consistent lens through which the heterogeneous behavior of different oilseed systems can be systematically compared and interpreted.

A central paradigm introduced in this review is the “Processing Window”, which reconciles the bidirectional functional effects observed across specialty oilseed systems. We posit that functional outcomes—whether the stabilization of an emulsion, enhancement of foaming capacity, or embrittlement of a gel—are not intrinsic properties of the interaction itself, but condition-dependent responses governed by binding density, polyphenol structure, and environmental factors such as pH, temperature, oxygen exposure, and the polyphenol-to-protein ratio. Within this window, moderate association (specific or non-specific) promotes beneficial structural reshaping that improves interfacial viscoelasticity, oxidative stability, and network formation. In contrast, excessive cross-linking triggers macromolecular collapse, masks bioactive sites, and reproduces the classic “oilseed problem” of technical and sensory deterioration (discoloration, aggregation, reduced solubility, and impaired digestibility) [8,11,12,13]. This review systematically evaluates the boundaries of this functional regime and provides practical guidance on how to navigate it, thereby enabling the precision valorization of oilseed-derived ingredients.

## 2. Polyphenol Diversity in Specialty Oilseeds: A Comparative Overview

For the present topic, the most informative comparison is not simply which crop contains more polyphenols, but how the dominant phenolics differ in size, polarity, oxidizability, and matrix accessibility. These features strongly influence whether a polyphenol mainly remains near the protein hydration layer, inserts into non-polar regions, or shifts toward oxidation-mediated covalent chemistry during processing [14,15,16].

Across the major specialty oilseeds discussed here, three questions are especially relevant: where the characteristic polyphenols are enriched (meal, hull, pellicle, pomace, or oil-associated fraction); whether they remain accessible during aqueous extraction and thermal processing; and whether their structures favor reversible association or extensive cross-linking. This comparison provides a more direct bridge from composition to function than crop background alone [14,17,18,19].

Overall, sunflower and olive are often discussed in relation to readily oxidizable phenolic acids or catechols, flaxseed and walnut in relation to sterically demanding lignans or tannins, sesame across a broader polarity continuum, and Camellia oleifera in relation to amphiphilic flavonoid glycosides. The practical consequence is that different crops are likely to differ in binding site accessibility, cross-linking tendency, and the concentration window that separates functional enhancement from over-aggregation.

Sunflower is dominated by chlorogenic acid (CGA), a relatively polar and readily oxidizable phenolic acid concentrated mainly in seeds and defatted meal [20,21,22,23]. Because CGA combines multiple hydrogen-bonding groups with an o-diphenol motif, sunflower systems are frequently used to illustrate the shift from reversible association under mild conditions to quinone-mediated covalent reactions under alkaline or oxidative processing. This makes sunflower a useful reference system for discussing both functional improvement and quality defects such as greening or browning.

Flaxseed differs in that its characteristic polyphenols are frequently associated with lignan-rich hull fractions, especially secoisolariciresinol diglucoside (SDG) and related complexes [24,25,26,27,28,29]. Their large size, glycosylation, and matrix association can limit accessibility, so the interaction behavior of flaxseed proteins often depends on extraction history and whether the phenolics are present as free molecules, enriched extracts, or native macromolecular assemblies. The reported effects therefore tend to emphasize structural rearrangement, delivery performance, and digestion behavior rather than rapid oxidation chemistry.

Sesame presents a broader polarity distribution, spanning lipophilic lignans in oil-rich fractions and more polar phenolics in defatted meal [30,31,32,33,34,35,36,37]. Small hydrophobic molecules such as sesamol may access non-polar protein regions more readily than bulky glycosides, whereas more polar phenolics are more likely to act near the hydration layer. Sesame is therefore useful for discussing how polyphenol size and polarity influence binding depth, interfacial behavior, and antioxidant performance.

Camellia oleifera meal is enriched in flavonoid glycosides and related phenolics that combine aromatic rings with sugar moieties [38,39,40,41,42,43]. This amphiphilic character suggests a dual interaction pattern, with aromatic regions contributing to hydrophobic contacts and glycosyl groups favoring hydrogen bonding at the protein–water interface. Available studies therefore often link Camellia oleifera phenolics to changes in wettability and Pickering-type interfacial assembly rather than to extensive oxidation-driven cross-linking.

Walnut is notable for ellagitannins and other tannin-rich pellicle components that are large, multidentate, and strongly associated with astringency [44,45,46,47,48]. Compared with CGA- or sesamol-dominant systems, these molecules are more likely to promote surface bridging, the dehydration of the protein interface, and aggregation or precipitation, especially when their concentration is high. At the same time, controlled processing may redirect part of this strong interaction toward textural applications.

Olive byproducts are characterized by oleuropein, hydroxytyrosol, and related secoiridoids or catechols concentrated in pomace and meal rather than the oil phase [49,50,51,52,53,54]. Because hydroxytyrosol can become highly reactive after oxidation, olive-based systems are frequently discussed in the context of covalent grafting, oxidative stability, and film or packaging applications. The evidence, however, remains stronger for selected formulated systems than for fully native olive protein matrices.

While crop identity provides essential context, the molecular behavior of protein–polyphenol systems in specialty oilseeds is more fundamentally governed by the chemical archetypes of their dominant endogenous phenolics. As summarized in Table 1, we classify these polyphenols into three primary structural–functional archetypes: (1) small, highly reactive phenolics (e.g., chlorogenic acid and hydroxytyrosol), characterized by low steric hindrance and high redox sensitivity, which readily enable deep residue-level binding and rapid transitions to covalent coupling; (2) bulky, glycosylated or sterically hindered phenolics (e.g., secoisolariciresinol diglucoside and flavonoid glycosides), which predominantly favor surface-mediated, non-specific interactions and interfacial reorganization; and (3) multidentate tannins (e.g., ellagitannins), which promote extensive protein bridging, dehydration, and macroscopic aggregation or precipitation. This comparative perspective resolves the inconsistencies in “interaction” outcomes across crops by linking structural parameters (MW and LogP) to specific binding modes.

## 3. Mechanisms of Protein–Polyphenol Interactions in Specialty Oilseeds

Protein–polyphenol interactions in specialty oilseeds are best interpreted as a dynamic continuum from reversible association to covalent coupling, rather than as two fully separate regimes. To resolve the bidirectional functional effects observed across diverse systems, we propose a “Processing Window” framework as a unifying conceptual model. Within this window, interactions are fundamentally categorized into specific interactions—characterized by stoichiometric binding to defined residue-level sites (linked to high binding affinity Ka)—and non-specific interactions, involving general surface-mediated adsorption driven by cumulative physical forces. Which regime dominates depends on polyphenol structure, protein composition, oxygen availability, pH, and the severity of processing [55,56,57].

**Figure 1:**
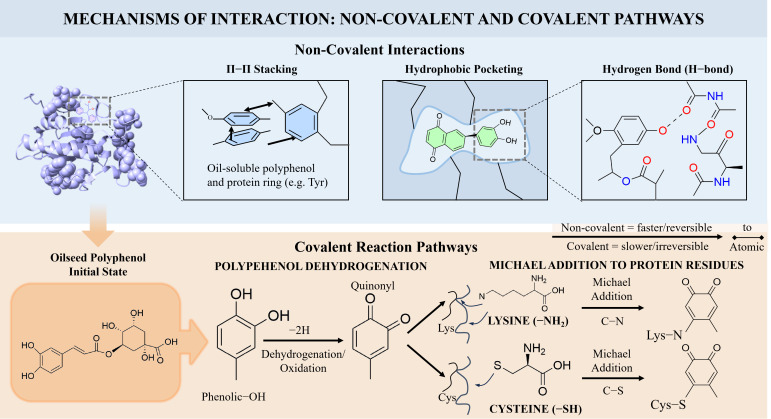
Microscopic schematic diagram of interaction mechanism.

Non-covalent association usually arises from hydrogen bonding, hydrophobic contacts, pi-pi interactions, and electrostatic effects. Covalent coupling becomes more likely when oxidizable polyphenols form quinones that react with nucleophilic amino acid side chains. In practice, many systems may pass from one regime to the other as processing conditions change, so mechanistic assignments should be made cautiously and ideally with converging evidence from multiple methods [10,55,56,57]. As illustrated by the microscopic mechanism model in Figure 1, the interaction network between proteins and polyphenols encompasses a bidirectional pathway from reversible physical adsorption to irreversible chemical bonding: under mild conditions, polyphenols primarily anchor to proteins via hydrogen bonding, hydrophobic intercalation, and π-π stacking (e.g., between the aromatic rings of polyphenols and tyrosine residues); conversely, under strong oxidation or alkaline induction, polyphenols rapidly dehydrogenate and convert into highly electrophilic quinone intermediates, which subsequently initiate nucleophilic attacks on lysine (-NH2) or cysteine (-SH) on the polypeptide chain, forming extremely robust covalent cross-links.

Functionally, the “Processing Window” defines the threshold between beneficial structural reshaping and detrimental quality collapse. Moderate interaction within this window can improve interfacial adsorption, oxidative protection, or network formation, whereas excessive association or over cross-linking may reduce solubility, limit enzyme accessibility, or intensify astringency and discoloration [9,11]. The frequently cited proposition that moderate binding is beneficial whereas over cross-linking is harmful is therefore useful as a working hypothesis, but its precise boundary depends on the protein type, polyphenol structure, oxidation degree, and the concentration window.

Across the six crop systems, a comparative pattern begins to emerge: small and oxidizable phenolics such as CGA or hydroxytyrosol can more readily switch between reversible binding and covalent chemistry, whereas bulky lignans, glycosides, and tannins more strongly reflect steric accessibility, multidentate binding, and matrix confinement. This comparison also highlights a major evidence gap, namely that detailed molecular claims are much better supported in sunflower and model-protein studies than in several other endogenous oilseed systems.

As shown in Table 2, the specific polyphenols enriched in sunflower, flaxseed, sesame, Camellia oleifera, walnut, and olive exhibit significant species differences in molecular polarity, steric hindrance, and lipid-water partition coefficients. The specificity of these underlying chemical structures directly determines their preferential binding sites, dominant physicochemical driving forces, and ultimate reaction pathways when interacting with macromolecular proteins.

### 3.1. Non-Covalent Interactions

Non-covalent interactions are the predominant driving forces in aqueous and partially hydrated oilseed systems. While usually discussed in terms of hydrogen bonding, hydrophobic association, and electrostatic attraction [76,77,78], these forces must be understood through the lens of binding specificity to predict functional outcomes.

We categorize these into two primary regimes: specific interactions, where certain small hydrophobic phenolics insert into defined non-polar residues or shallow protein pockets with relatively high binding affinities (Ka) and stoichiometric control, and non-specific interactions, involving general surface-mediated adsorption with weaker affinities and lower stoichiometry. For example, sesamol interacting with whey proteins (WPI/β-LG) in model systems exhibits non-specific binding, characterized by low binding affinity (KD approximately 1.75 × 10^−3^), very small stoichiometric number (*n* = 1.6 × 10^−2^), and primary driving forces of hydrogen bonding and van der Waals interactions at the protein surface cavity rather than deep hydrophobic pockets [33]. Nevertheless, findings from such simplified model systems (e.g., purified whey proteins) must be distinguished from complex endogenous food matrices, where competing components may mask or alter these interfacial sites.

In specialty oilseeds systems, this structure-dependent behavior is more evident through the contrast between “anchoring” and “bridging”. Amphiphilic Camellia oleifera phenolics, acting as specific/surface-active binders, favor interfacial rearrangement by anchoring through dual hydrophobic and hydrogen bonding. Conversely, bulky walnut ellagitannins behave as non-specific multidentate binders that promote surface bridging, dehydration, and macroscopic aggregation. This distinction confirms that binding depth, governed by polyphenol size and residue accessibility, is the root determinant of whether the non-covalent state remains reversible or progresses toward the quality deterioration thresholds defined in the “Processing Window” [68,69,79,80].

### 3.2. Covalent Interactions

Covalent interactions represent the irreversible end of the protein–polyphenol interaction continuum, characterized by stable, high-density covalent modifications at specific nucleophilic amino acid residues. These interactions are generally triggered when oxidizable polyphenols are oxidized to form highly reactive quinones (or quinone methides), which then undergo nucleophilic addition by amino acid side chains, primarily lysine (Lys), cysteine (Cys), and histidine (His) [57,81].

Three main routes are commonly discussed for inducing covalent polyphenol–protein interactions: enzyme-mediated oxidation, free-radical grafting, and alkaline auto-oxidation. Olive-derived hydroxytyrosol is frequently cited as a representative catechol that readily participates in quinone chemistry. Nevertheless, the rate and extent of covalent grafting remain highly dependent on oxygen exposure, enzyme activity, and the availability of nucleophilic sites on the protein [82,83].

Free-radical grafting has been reported in model and modified protein systems, including flaxseed-related studies [62,84,85]. These results show that phenolic compounds can be covalently incorporated into protein networks under redox-initiated conditions. However, the relevance of such deliberately activated systems to spontaneous transformations in real food matrices should be clearly differentiated.

Alkaline treatment is particularly important because it can drive the transition from an initially non-covalent system toward oxidation-mediated covalent cross-linking. Studies on sunflower chlorogenic acid (CGA) provide some of the clearest evidence for this shift, often accompanied by marked structural changes and visible greening or browning. Nevertheless, the critical pH threshold for this transition should not be assumed to be identical across all oilseed matrices [8,11,59,86].

### 3.3. Influencing Factors

The extent and pathway of protein–polyphenol interactions are strictly governed by the processing environment, which acts as the “Window Regulator” for functional outcomes. Rather than acting independently, key variables such as pH, temperature, and the polyphenol-to-protein concentration ratio act synergistically to position the system within the moderate interaction regime [87].

pH and Oxidative Conditions: These serve as the primary switches for the transition from non-covalent to covalent regimes. Neutral or mildly acidic environments generally maintain the system within the beneficial non-covalent zone, whereas alkaline conditions (often exceeding pH 8.0–9.0 in sunflower systems) and high oxygen exposure trigger the formation of reactive quinones, pushing the system toward the detrimental over cross-linking zone [47,79,88,89].

Temperature and Ionic Strength: These factors modulate the “structural accessibility” of the protein. Moderate thermal treatment combined with appropriate ionic strength enhances protein–polyphenol interactions by simultaneously exposing buried hydrophobic pockets and screening electrostatic repulsions, thereby facilitating the closer approach of the polyphenols to the protein surface. However, exceeding a critical threshold—whether through excessive heating or very high ionic strength—leads to extensive denaturation and macromolecular collapse, effectively “closing the window” for productive functional binding and favoring non-specific aggregation instead [90,91].

Concentration Ratio: This defines the stoichiometric limit of the interaction. An optimal intermediate ratio typically enables targeted residue-level anchoring and productive binding, whereas the supersaturation of polyphenols promotes excessive intermolecular bridging and electrostatic shielding, ultimately resulting in macroscopic phase separation—the phenomenon commonly referred to as “the oilseed problem” [20,92].

Crucially, while these qualitative trends are established, the quantitative boundaries—the exact pH value or concentration threshold at which functionality shifts—remain largely undefined for most endogenous specialty oilseed matrices. Addressing this knowledge gap is essential for transitioning from descriptive observations to the precision design of functional plant-based systems.

## 4. Characterization Methods of Interactions

**Figure 2:**
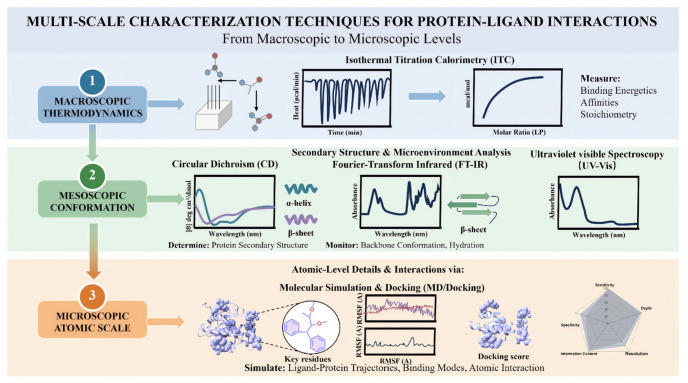
Flowchart of multi-scale characterization technology.

Because protein–polyphenol interactions in specialty oilseeds are multistep and condition-dependent, no single analytical method can fully identify binding mode or functional consequence. The current evidence is strongest when spectroscopic, thermodynamic, and computational results are interpreted within a multiscale analytical paradigm, provided that measurements from homologous oilseed systems are strictly distinguished from those derived from simplified model proteins (e.g., BSA or whey proteins). As illustrated in Figure 2, modern food science has established a cross-scale comprehensive analytical system, encompassing macroscopic thermodynamic energy exchanges (e.g., determining binding affinity via ITC), mesoscopic macromolecular secondary structure and microenvironmental remodeling (e.g., UV-vis, FT-IR, CD), down to microscopic atomic-level spatial topology and dynamic trajectory tracking (e.g., MD simulations and molecular docking). Such a holistic approach is essential to capture the “residue-level mapping” required to navigate the processing windows of endogenous oilseed matrices and to distinguish specific from non-specific binding states.

### 4.1. Experimental Spectroscopic Characterization

Experimental spectroscopic methods provide essential insights into chromophore environments and secondary structure remodeling. However, for specialty oilseeds, their primary value lies in narrowing plausible mechanisms rather than providing stand-alone proof of specific binding sites [88,93,94].

#### 4.1.1. UV-Vis

Ultraviolet-visible (UV-vis) spectroscopy is a fundamental optical technique for investigating polarity alterations in the macromolecular microenvironment. Its physical mechanism is based on the energy level transitions of conjugated π electrons in aromatic amino acid residues (e.g., tryptophan and tyrosine) within the protein polypeptide chain upon absorbing radiation of specific wavelengths [95]. When specialty oilseed polyphenols undergo physical intercalation or chemical cross-linking with proteins, the solvation shell and local dielectric constant surrounding these chromophores are significantly altered, leading to a red shift or blue shift in the characteristic absorption peak, accompanied by marked fluctuations in the extinction coefficient [22,96].

Within this analytical framework, UV-vis serves as an important tool for distinguishing between reversible association and the transition to specific covalent coupling. Taking the sunflower macromolecular system as an example, Jia et al. investigated the alkaline-mediated interactions between sunflower protein isolate (SFPI) and chlorogenic acid (CGA). They employed reversed-phase high-performance liquid chromatography (RP-HPLC) at 330 nm to monitor changes in the content of free CGA and protein-bound CGA. Concurrently, through solution UV-vis spectroscopy in the visible region (400–800 nm), they observed that, under alkaline conditions (pH 9) that induced covalent binding, when the CGA-to-protein molar ratio reached ≥ 1:1, the solutions gradually turned green, accompanied by the appearance of a new characteristic absorption peak at approximately 700 nm. The absorbance of this peak increased significantly with the progress of the reaction. This optical response confirms, from the perspective of molecular chromophores, that after chlorogenic acid is oxidized to o-quinone, it undergoes irreversible Michael addition reactions with nucleophilic groups on the protein side chains (such as Lys and Cys), thereby forming C-N covalent cross-linking networks with a strong conjugated system [11].

UV-vis is highly effective for monitoring browning kinetics and the onset of covalent coupling, chromophore perturbation data in complex oilseed matrices should be interpreted as indirect evidence. Due to potential signal overlap from endogenous pigments, these optical responses alone cannot fully resolve the binding affinity or the secondary structure alterations. To achieve a high-dimensional characterization of this interaction landscape, UV-vis findings must be validated through a multiscale analytical approach: this includes thermodynamic profiling via ITC to quantify binding constants Ka and conformational analysis via FT-IR and CD to monitor the redistribution of α-helices and β-sheets. Such convergent evidence is essential to confirm whether the interaction remains within the functional enhancement zone or has triggered irreversible aggregation.

#### 4.1.2. FT-IR

Fourier transform infrared (FT-IR) spectroscopy not only serves as a classical vibrational technique for the quantitative assessment of protein topological transitions but also functions as a versatile tool for identifying the chemical identity of matrix-associated components, such as mucilage and lignans. While its analytical strength is predominantly centered on the amide I band (1600–1700~cm^−1^) [97,98]. which reflects the hydrogen-bonding networks of the peptide backbone, its application in specialty oilseed research is further enhanced by the fingerprint region (1200–700~cm^−1^). Within this analytical context, FT-IR provides a multi-dimensional perspective to distinguish between global structural remodeling and the co-existence of endogenous components during extraction and modification [88,99].

In flaxseed systems, FT-IR and fluorescence spectroscopy were first employed to examine protein fractions (WEF, SEF, and AEF) sequentially extracted from defatted flaxseed meal. While all fractions shared characteristic signals of peptide backbones, significant differences were observed in the fingerprint region (1200–750~cm^−1^), associated with mucilage polysaccharides, where WEF exhibited the strongest intensity. Notably, the C=O vibrations within the 1600–1750~cm^−1^ range (attributed to amide and ester groups) confirmed the co-extraction of phenolic compounds (e.g., SDG and phenolic acids), particularly in the alkaline-extracted AEF [99]. Further systematic investigations revealed that, compared to non-covalent mixtures, covalent conjugates of flaxseed protein isolate (FPI) prepared via free-radical or alkaline methods undergo more profound conformational reorganization. Quantitative fitting of the amide I band (600–1700~cm^−1^) showed that covalent modification nearly doubled the random coil content while significantly depleting β-sheets, indicating a transition from an ordered to a disordered state. These structural changes—driven by non-covalent anchoring and covalent nucleophilic addition—provide a molecular explanation for the enhanced interfacial dilatational elasticity and oxidative stability observed in specialty oilseed protein-based emulsions [100].

While FT-IR is invaluable for tracking ligand co-existence and ensemble protein remodeling within the established interaction domain, it primarily captures average structural redistribution. Spectral shifts in the amide or fingerprint regions confirm that a conformational change has occurred but lack the resolution to uniquely pinpoint specific binding residues. Consequently, to achieve the “residue-level mapping” target, these FT-IR observations must be cross-validated with circular dichroism (CD) to detect fine-scale backbone chirality and isothermal titration calorimetry (ITC) to quantify precise thermodynamic affinities (Ka).

#### 4.1.3. CD

Compared to infrared spectroscopy, far-ultraviolet circular dichroism (far-UV CD, 190–250 nm) detects the unequal absorption of left- and right-circularly polarized light by the chiral polypeptide backbone. Utilizing the characteristic CD signal spectra formed by different secondary structures (e.g., α-helices, β-sheets), this technique offers a more sensitive reflection of changes in protein conformation [101]. This sensitivity makes CD particularly effective for monitoring protein folding/unfolding processes and ligand-binding effects within this functional scope. Beyond the far-UV region, near-UV CD (260–320 nm) further provides a fingerprint for the asymmetric environment of aromatic residues, thereby mapping the tertiary structural remodeling of the protein matrix that vibrational spectroscopy might overlook.

In the walnut macromolecular system, researchers employed CD spectroscopy to investigate the interaction between endogenous polyphenols from walnut pellicle (primarily ellagitannins and ellagic acid derivatives) and walnut protein isolate (WalPI) or walnut globulin [71,80,102]. Spectral deconvolution and secondary structure analysis revealed that these polyphenols, acting through extensive hydrogen bonding and hydrophobic interactions, induce significant conformational unfolding of the walnut storage proteins. Specifically, the binding leads to a marked decrease in ordered secondary structures—particularly β-sheet content—and a corresponding increase in disordered random coil structures. These transitions are accompanied by tertiary structural changes, as evidenced by fluorescence quenching and shifts in the microenvironment of aromatic amino acid residues. Such structural shifts, further supported by scanning electron microscopy and surface hydrophobicity data, provide a molecular-level explanation for modified functional properties, including enhanced foaming capacity and reduced allergenicity. CD thus provides direct spectroscopic evidence for how intense non-covalent interactions disrupt the compact native conformation of woody oilseed globulins.

While CD is peerless for detecting global chirality shifts and secondary structure redistribution, it remains a qualitative tool regarding the precise location of binding sites. In complex oilseed matrices, CD signals can be sensitive to local dielectric fluctuations that do not always correlate linearly with macroscopic functional changes. Therefore, to achieve “residue-level mapping,” CD findings must be integrated with isothermal titration calorimetry (ITC) to resolve the thermodynamic signatures and stoichiometric ratios (n) of the protein–polyphenol complexes.

#### 4.1.4. ITC

Isothermal titration calorimetry (ITC) is an absolutely quantitative technique for the direct determination of thermodynamic parameters of macromolecule-ligand binding. Unlike spectroscopic methods that monitor conformational transitions, ITC directly measures the heat exchange (q) during the titration process, allowing for the simultaneous determination of binding affinity (Ka), stoichiometry (*n*), and enthalpy change (△H). These parameters facilitate the calculation of Gibbs free energy (△G) and entropy change (△S) [103,104], thereby revealing the fundamental molecular driving forces—such as hydrogen bonding, van der Waals forces, or hydrophobic effects—that govern the complex formation within this thermodynamic space.

The interaction between sesamol and whey protein isolate (WPI) offers a representative model for assessing the interfacial thermodynamics of phenolic compounds. ITC titration curves demonstrate that sesamol binds spontaneously to WPI (△G < 0) via an exothermic process (△H < 0) characterized by a negative entropy change (△S < 0). However, the equilibrium dissociation constant (KD = 1.75 × 10^−3^ M) and the notably low stoichiometric number (*n* = 1.6 × 10^−2^) suggest that this interaction is relatively weak and predominantly non-specific. Driven primarily by hydrogen bonding and van der Waals forces, this non-covalent adsorption allows sesamol to anchor onto the interfacial proteins, thereby explaining the enhanced physical and oxidative stability of WPI-stabilized fish oil emulsions [33].

While ITC provides an absolute quantitative measure of interaction energetics in purified model systems, its application to real, endogenous oilseed matrices is fraught with methodological challenges. The technique is highly sensitive to the heat of dilution and non-specific heat signals generated by heterogeneous components (e.g., residual lipids or polysaccharides) in crude extracts. Consequently, thermodynamic parameters from model studies like the WPI–sesamol system must be interpreted cautiously when applied to native oilseed globulins. Achieving a true “residue-level mapping” requires integrating these thermodynamic findings with MD simulations to provide the atomic-scale context for the observed energy changes.

### 4.2. Computational Biology Simulations

Computational biology simulations provide a high-resolution digital lens to decipher molecular interactions at the atomic scale, effectively bridging the resolution gap between macroscopic thermodynamic observations and mesoscopic structural changes. These tools allow for the visualization of the spatial topology and dynamic trajectories of protein–polyphenol complexes, offering molecular-level insights into this conformational landscape.

#### 4.2.1. Molecular Docking

Molecular docking serves as a predictive tool for identifying preferential binding sites and evaluating the binding affinity of polyphenols within the protein matrix. By searching for optimal binding poses and calculating scoring functions, this methodology generates hypotheses regarding the residue-level specificity of interactions, providing a theoretical baseline for experimental validation [105].

This study investigated the binding modes between representative walnut pellicle polyphenols, such as corilagin, and the major storage proteins (11S and 7S globulins) of walnut protein isolate. The docking results revealed favorable binding free energies (△G ranging from −5.2 to −8.8 kcal/mol), demonstrating that ellagitannin-type polyphenols form extensive hydrogen-bonding networks with polar residues, including ASN240, GLN239, and SER243. These ligands simultaneously engage in hydrophobic interactions and a diverse array of π-type interactions with specific amino acid side chains such as ARG270, ASP267 and GLU. Such atomic-level insights provide rigorous mechanistic support for the polyphenol-induced secondary structure rearrangement observed via FT-IR spectroscopy, characterized by a decrease in β-sheet content and a corresponding increase in random coil structures. These findings further elucidate the marked reduction in surface hydrophobicity and the resulting conformational unfolding, thereby accounting for macroscopic functional improvements such as enhanced water-holding and foaming capacities [80].

Despite its predictive power, the accuracy of molecular docking in specialty oilseed research is often limited by the lack of high-resolution crystal structures for endogenous globulins. To address this, the integration of Homology Modeling is essential to construct reliable protein templates. Transitioning from generic model proteins to homology-modeled native structures ensures that the simulated hydrophobic cavities and electrostatic surfaces accurately represent the unique microenvironments of oilseed-derived macromolecules.

#### 4.2.2. Molecular Dynamics Simulation

Molecular dynamics (MD) simulations extend beyond the static “snapshots” provided by docking to capture the temporal stability and conformational evolution of protein–polyphenol complexes. By integrating Newtonian mechanics with atomic-scale force fields, MD trajectories record the real-time redistribution of molecular forces within the “Processing Window”. Key parameters, including root-mean-square deviation (RMSD) and root-mean-square fluctuation (RMSF), serve as quantitative indicators of global stability and residue-specific flexibility, respectively. Furthermore, tracking the radius of gyration (Rg) and solvent-accessible surface area (SASA) offers a microscopic perspective on how phenolic ligands induce the compaction or unfolding of the protein matrix [88,106]. These metrics effectively validate the conformational transitions previously observed via FT-IR or CD spectroscopy. In specialty oilseed research, MD simulations are instrumental in elucidating whether a specific polyphenol—such as chlorogenic acid or corilagin—acts as a “molecular wedge” that triggers protein denaturation or a stabilizing agent that reinforces the polypeptide network.

A compelling example is found in the myosin system, where the synergistic application of molecular docking and MD simulations revealed that sesamol anchors to myosin primarily through hydrophobic interactions and hydrogen bonding. These interactions drive the formation of a more compact and stabilized protein architecture, as evidenced by the convergence of MD trajectories. Functionally, this structural transition effectively reduces the surface hydrophobicity of the sesamol–myosin complex, thereby directly improving its solubility and emulsification properties. Such findings provide rigorous mechanistic evidence for the potential application of sesamol as a functional modulator in meat protein systems, illustrating how atomic-scale stabilization translates into enhanced macroscopic performance [66].

Despite offering high-resolution insights, MD simulations remain inherently model-dependent and computationally intensive. The accuracy of predicted trajectories is confined by the selection of force fields and the simplification of the solvent environment, which may not fully replicate the complexity of an endogenous oilseed-based food matrix. Consequently, MD results achieve their maximum scientific validity when they demonstrate convergence of evidence with experimental thermodynamic data and spectroscopic findings, ensuring that digital hypotheses align with physical reality.

### 4.3. Comparative Interpretation of Characterization Tools

In practice, UV-vis is most useful for following chromophore changes and oxidation-related shifts, FT-IR and CD are employed to monitor secondary structure trends and global folding/unfolding. ITC provides an absolute quantitative description of binding thermodynamics, and molecular docking or dynamics simulations are instrumental for proposing residue-level binding scenarios. However, none of these methods alone can unambiguously distinguish non-covalent from covalent events in complex food matrices. The most reliable interpretation therefore combines at least one structural method, one thermodynamic or compositional method, and clear information on processing conditions, especially when conclusions are transferred from whey, BSA, or beta-LG models to endogenous specialty-oilseed systems. Table 3 summarizes the major analytical roles and their main limitations.

## 5. Effects of Interactions on Polyphenols and Proteins

The bridge between molecular-scale assembly and macroscopic functional performance is the central nexus of protein–polyphenol research in specialty oilseeds. While the preceding sections elucidated the multiscale mechanisms of binding, this section establishes a rigorous “structure–interaction–function” mapping chain to explain the divergent physicochemical outcomes observed during processing. Central to this analysis is the “Processing Window” framework, which dictates that functional attributes are not merely a function of polyphenol concentration, but are determined by the precision of the binding mode—ranging from residue-specific covalent anchoring to non-specific, multidentate surface interactions. By critically evaluating how these diverse assembly pathways remodel the protein matrix, this review moves beyond phenomenological descriptions to provide a mechanistic basis for tailoring the solubility, interfacial activity, and stability of oilseed-derived functional ingredients. This approach allows for a clearer distinction between synergistic structural refinement and the deleterious consequences of excessive molecular cross-linking, providing a conceptual roadmap for the development of high-performance plant-based food systems.

### 5.1. Solubility and Thermal Stability

Solubility serves as a fundamental indicator of the physicochemical state of protein–polyphenol complexes, as it directly reflects changes in surface hydrophobicity (H_0_) and the net charge distribution of the macromolecular matrix. Within this moderate assembly window, the modulation of solubility is governed by the transition between localized molecular anchoring and ensemble-level structural aggregation [76,107,108]. While moderate non-covalent interactions can stabilize proteins by increasing steric hindrance or modifying the solvation shell, excessive binding—particularly through non-specific pathways—often triggers the burial of hydrophilic residues and the formation of insoluble clusters. However, excessive binding—particularly through non-specific pathways—often triggers the burial of hydrophilic residues and the subsequent formation of insoluble clusters [88]. Similarly, thermal stability is determined by the cooperative nature of polypeptide folding, which phenolic ligands can either reinforce or disrupt depending on their binding density and site specificity [76].

A clear illustration of the transition from beneficial modification to detrimental macroscopic collapse is provided by the interaction between endogenous walnut pellicle polyphenols (WPP) and walnut protein isolate (WalPI). Dominated by bulky, multidentate ellagitannins, WPP interact with WalPI primarily via a non-specific, surface-mediated pathway. These large polyphenols function as “molecular bridges,” establishing extensive hydrogen-bonding networks and hydrophobic contacts across multiple surface sites without strict residue-level specificity. Multiscale evidence supports this non-specific assembly mechanism: FT-IR analysis reveals a marked shift from ordered β-sheets to disordered random coils and α-helices; surface hydrophobicity (H_0_) decreases by 62.9% as the polyphenols mask the hydrophobic pockets of the protein. Furthermore, differential scanning calorimetry (DSC) records a significant reduction in peak denaturation temperature (Tp) from 89.97 °C in dephenolized WalPI to 85.95 °C in the polyphenol-rich system. This decline, accompanied by altered enthalpy of denaturation, indicates that the non-specific adsorption of bulky ellagitannins acts as a “molecular wedge,” disrupting the internal stabilizing forces of the globulins and promoting premature thermal unfolding. Macroscopically, this structural remodeling results in a significant functional trade-off: while the water-holding capacity increases from 1.72 to 2.48 g/g due to an expanded hydrophilic surface, the overall solubility, oil-holding capacity, and thermal stability suffer a severe decline as a result of irreversible aggregate formation [80].

When assessing solubility and thermal stability, a clear distinction must be maintained between purified model systems and real oilseed matrices. In crude extracts, the presence of endogenous mucilage or lipids can interfere with polyphenol–protein binding, potentially shielding the protein from the aggregation observed in purified systems. Therefore, the “molecular bridge” effect identified in the walnut system should be interpreted as a potential functional bottleneck that requires precise control of polyphenol removal or encapsulation to maintain protein functionality in industrial applications.

### 5.2. Interfacial Properties: Emulsification and Foaming

The interfacial behavior of specialty oilseed proteins—encompassing their ability to adsorb and stabilize air–water or oil–water interfaces—is profoundly reshaped by polyphenol-mediated interactions [109]. These functional shifts generally stem from moderate protein unfolding, calibrated surface hydrophobicity, and the formation of thicker, more viscoelastic interfacial layers. Within the “Processing Window,” phenolic ligands can act as molecular anchors that reinforce the interfacial film, thereby preventing droplet coalescence or foam drainage. However, the same variables can impair performance if the resulting complexes become too rigid, overly large, or insufficiently soluble for rapid migration and adsorption at the interface. The transition from functional enhancement to impairment is dictated by the binding mode, where specific covalent anchoring often provides superior structural stability compared to reversible non-covalent association [76,110].

A representative instance of functional optimization through the covalent pathway is the grafting of tea polyphenols (TP) and flaxseed polyphenols (FP) onto flaxseed protein isolate (FPI). Under alkaline conditions (pH 9.0) that fall within the moderate interaction window, phenolic hydroxyl groups are oxidized into electrophilic quinone intermediates, which subsequently undergo Michael addition or Schiff-base reactions with nucleophilic residues—primarily the ε-NH_2_ of lysine and -SH of cysteine—on FPI. This covalent anchoring, supported by a grafting efficiency of up to 29.68%, triggers a significant secondary structure rearrangement, characterized by a transition from ordered β-sheets to disordered random coils. Multiscale evidence, including new UV-vis absorption peaks and fluorescence red shifts, confirms the formation of stable C–N and C–S cross-links. Macroscopically, these covalent conjugates (notably FPI-TP-A) exhibit markedly enhanced emulsifying activity and stability, alongside improved foaming capacity. Furthermore, the antioxidant activity of the system is dramatically elevated, with radical scavenging capacities increasing 6–112-fold compared to native FPI. This demonstrates a clear positive structure–function linkage where controlled covalent grafting reinforces the interfacial barrier while endowing it with potent radical-quenching capabilities [100].

The flaxseed example underscores the importance of maintaining an optimal reaction scope to avoid the detrimental consequences of over cross-linking. While specific covalent grafting can maximize interfacial rigidity and antioxidant protection, excessive oxidation risks the formation of insoluble aggregates that cannot rapidly reach the interface, leading to a decline in emulsifying efficiency. Moreover, a critical distinction must be made between purified conjugates and real oilseed matrices. In authentic flaxseed systems, the presence of endogenous mucilage may competitively interact with polyphenols or interfere with the grafting kinetics at the oil–water interface. Therefore, future research must focus on defining the precise pH and concentration thresholds required to replicate these laboratory-scale functional gains in complex, multi-component food environments without compromising sensory attributes or digestibility.

### 5.3. Nutritional Attributes

The nutritional profile of specialty oilseed proteins is fundamentally reshaped by the chemical identity and binding modes of associated polyphenols within the “Processing Window.” These interactions create a delicate balance between chemical protection and metabolic availability: while phenolic hydroxyl groups preserve high radical scavenging capacity and shield proteins from oxidation, overly strong coupling or excessive aggregation can mask cleavage sites and restrict enzymatic access [9,89].

In flaxseed systems, non-covalent complexation with endogenous glycosylated phenolics (such as secoisolariciresinol diglucoside and ferulic acid derivatives) exemplifies the beneficial side of this balance. Fluorescence spectroscopy reveals a ~50% reduction in tryptophan fluorescence intensity accompanied by a red shift in emission maxima from 348 nm to 356 nm, while principal component analysis confirms that the protein matrix primarily donates H^+^, whereas the incorporated phenolics enable a dual-action mechanism involving both electron donation and H^+^ transfer. This synergy results in up to an 80% increase in ORAC and FRAP values, as protein unfolding exposes additional nucleophilic sites while the bound phenolics retain their full antioxidant potency [61].

However, in the walnut system, endogenous walnut pellicle polyphenols (WPP) exert a marked digestion-retardation effect on walnut protein isolate (WalPI). Structural studies show that WPP induce partial unfolding of WalPI, characterized by a decrease in β-sheet content and an increase in random coil structures, accompanied by reduced surface hydrophobicity and solubility [80]. More critically, walnut pellicle extracts (WPE) and its predominant monomeric polyphenols (especially epigallocatechin gallate (EGCG), followed by chlorogenic acid, (+)-catechin, and ellagic acid) strongly inhibit the activities of key digestive enzymes pepsin and trypsin. This inhibition, confirmed by fluorescence quenching, surface plasmon resonance (SPR), molecular docking and Michaelis–Menten kinetics, significantly lowers the in vitro digestibility of WalPI during both simulated gastric and intestinal phases. The net result is a pronounced reduction in the degree of hydrolysis of walnut protein–polyphenol complexes in the upper gastrointestinal tract, primarily driven by enzyme–polyphenol binding rather than direct protein compaction [111].

Interestingly, this apparent trade-off can be harnessed to improve the bioaccessibility of lipophilic payloads. Synergistic co-assembly can effectively overcome the low bioavailability of lipophilic substances. In flaxseed protein–polyphenol (FPI-FPP) nanocomposite emulsions, the interfacial complex layer significantly improves the stability of zeaxanthin dipalmitate (ZD). Compared to FPI-only systems, the FPI-FPP layer reduces droplet aggregation and ensures markedly higher ZD retention throughout gastrointestinal transit. Consequently, the bioaccessibility of ZD reaches 46.52%, a substantial improvement over the 26.86% observed in native FPI emulsions. This gain results from the dual effects of FPP antioxidant protection and the enhanced structural integrity of the interfacial membrane that facilitates micellization [112].

Collectively, these findings highlight an “Antioxidant-Digestibility Paradox”: protein–polyphenol complexation can simultaneously maximize oxidative stability and bioaccessibility of guest bioactives while imposing a functional penalty on the upper-gastrointestinal absorption of the protein itself. Precisely controlling interaction intensity within the moderate “Processing Window” is therefore essential. Moreover, most current evidence derives from static in vitro models that may not fully capture the dynamic feedback loops and microbial transformations occurring in the human gut. Future studies should focus on identifying optimal concentration thresholds that balance chemical protection with nutritional digestibility in complex food matrices.

### 5.4. Quantitative Landscape and Critical Thresholds

The transition from phenomenological observation to predictive control requires a rigorous quantitative mapping of the protein–polyphenol landscape. As established in the preceding sections, functional outcomes are not randomized; they are governed by specific thermodynamic and environmental thresholds, primarily pH, temperature, and the polyphenol-to-protein mass ratio. Identifying these “critical thresholds” allows for the precise demarcation of the “Processing Window,” where beneficial structural refinement can be achieved without triggering macroscopic collapse.

To provide a systematic overview of the interaction landscape, Table 4 synthesizes the core quantitative parameters across major specialty oilseed systems. This comparative synthesis highlights that while herbaceous systems (e.g., sunflower) are highly sensitive to pH-driven covalent thresholds, woody systems (e.g., walnut) are often limited by the steric and stoichiometric constraints of bulky multidentate ligands.

A cross-system comparison of the quantitative landscape synthesized in Table 4 reveals that the molecular behavior of specialty oilseed complexes is governed by predictable thermodynamic and environmental clustering. For non-covalent specific interactions, the apparent binding affinity reported in the table (Ka = 1.44 × 10^5^ M^−1^ for sesamol in the sesame system) falls within the typical range observed for localized hydrophobic pocketing and directed hydrogen-bonding networks. Crucially, the transition across the “Processing Window” boundary exhibits two distinct archetypal threshold behaviors. Small, redox-sensitive phenolics (such as sunflower chlorogenic acid) obey a sharp, pH-dependent step-threshold, remaining reversibly non-covalent at neutral or mildly acidic conditions but undergoing an abrupt irreversible transition to covalent Michael addition networks when the CGA:protein molar ratio reaches ≥1:1 at pH 9.0. Conversely, bulky, sterically hindered, or multidentate systems (such as walnut ellagitannins, flaxseed SDG, and Camellia oleifera glycosides) display a stoichiometry-driven mass ratio threshold. In these matrices, the transition from functional structural reshaping to macroscopic phase separation (aggregation or embrittlement) is relatively independent of pH and is triggered when the local polyphenol-to-protein ratio or content exceeds a critical saturation point (e.g., Camellia oleifera encapsulation at 3% COSCP), forcing extensive intermolecular bridging and surface dehydration. Mapping these bifurcated threshold behaviors provides a practical baseline for predicting ingredient stability and guiding precision processing of specialty oilseeds.

While the established thresholds provide a preliminary roadmap, a significant “knowledge gap” remains regarding the transferability of these findings to complex industrial environments. Most current data are derived from purified model systems, which may fail to capture the “buffer effects” of endogenous lipids or polysaccharides present in real oilseed matrices. Furthermore, the “Antioxidant-Bioavailability Paradox” suggests that the optimal window for chemical stability often conflicts with the window for nutritional digestibility. This misalignment underscores the necessity for multi-objective optimization. Future research must utilize surface response methodology (RSM) or machine learning to map the multi-dimensional interplay between processing variables, thereby defining the true industrial boundaries for the high-value utilization of specialty oilseeds.

### 5.5. Limitations of In Vitro Models and Implications for Real Food Matrices and Industrial Applications

The vast majority of mechanistic insights regarding protein–polyphenol assembly are derived from simplified, highly dilute in vitro model systems using homologous proteins such as bovine serum albumin (BSA) or whey isolates. While these models provide high-resolution thermodynamic and spectroscopic baselines, they fundamentally lack the macromolecular crowding effects inherent to authentic food systems [116]—effects that alter excluded volume, effective ligand concentration, and protein conformational equilibria in ways that cannot be captured by dilute binary systems. In an unpurified, native specialty oilseed matrix (such as full-fat or partially defatted sunflower meal, flaxseed, or soybean), the co-presence of lipids, carbohydrates, minerals, and other biomolecules gives rise to polyphenol–protein binding dynamics that are substantially more complex than those observed in isolated or purified protein model systems [117]. These multi-component environments, as encountered in real food processing and native matrices, can modulate protein conformation, alter binding site accessibility, and collectively influence the functional and nutritional properties of both proteins and phenolics [118]. Consequently, binding parameters derived from simplified bench-top models using isolated proteins may not fully capture the interaction dynamics of the native matrix, where additional co-solutes and processing conditions collectively perturb both the thermodynamic driving forces and the conformational remodeling kinetics of polyphenol–protein assembly.

A primary barrier to the direct translation of in vitro data is the “structural buffer effect” exerted by non-proteinous biopolymers and lipid bodies co-existing within crude oilseed streams. As detailed in Table 4, while a purified flaxseed or sunflower protein isolate interacts predictably with phenolic acids, real-world industrial extracts contain substantial fractions of endogenous mucilage polysaccharides, hull fibers, and residual neutral lipids. These macromolecular fractions act as competitive sinks for polyphenols: amphiphilic phenolics can migrate to the lipid–water interface, where they are sequestered through hydrophobic co-partitioning with residual neutral lipids rather than engaging protein binding sites; simultaneously, glycosylated phenolic constituents bind directly to polysaccharide networks via non-specific hydrogen bonding and hydrophobic bridging interactions, a process further compounded by the steric hindrance imposed by the glycosyl moiety, which concurrently reduces the polyphenol’s affinity for protein hydrophobic pockets [117,119,120,121]. Taken together, these competing adsorption pathways effectively shield the protein’s hydrophobic cavities from the intended polyphenol–protein interaction [118]. Consequently, industrial processors cannot assume identical grafting efficiencies or conformational remodeling kinetics as observed in crystalline or highly purified model configurations.

## 6. Food Applications

**Figure 3:**
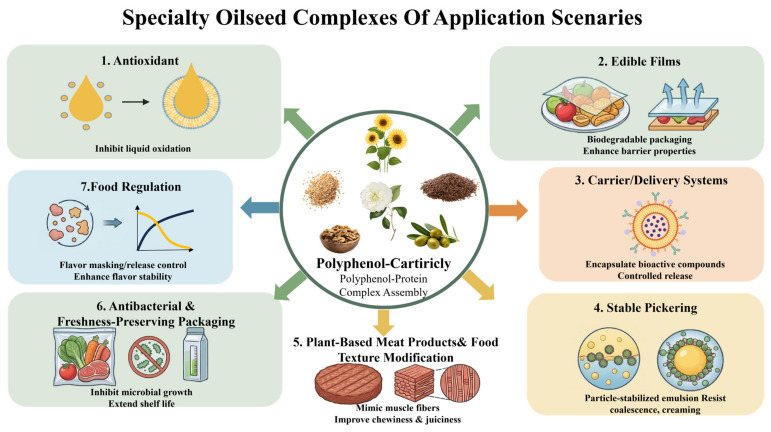
Industrial application scenarios of specialty oilseed complexes.

The ultimate objective of protein–polyphenol research in specialty oilseeds is the precise translation of molecular-scale assembly into macroscopic food system functionality. This transition is conceptualized through a rigorous “structure–interaction–function” mapping chain, which serves as the theoretical blueprint for the precision design of functional ingredients. As illustrated in Figure 3, the controlled assembly of specialty oilseed complexes provides a versatile engineering toolkit for addressing technical bottlenecks across seven core application scenarios: (1) antioxidant systems for inhibiting lipid oxidation; (2) edible films for sustainable packaging; (3) carrier/delivery systems for controlled bioactive release; (4) stable Pickering emulsions with high resistance to coalescence; (5) plant-based meat products for fiber mimicking; (6) antibacterial packaging for shelf life extension; and (7) food regulation for flavor masking and stability. This functional translation is strictly governed by the “engineering tolerance,” which defines the optimal balance between synergistic structural refinement and the avoidance of sensory or nutritional deterioration.

To provide a systematic evaluation of these functional gains, the advanced applications are categorized into three strategic domains: (1) fluid-interfacial and delivery systems, (2) solid-state matrices and active packaging, and (3) structural texturization and sensory modulation. Table 5 provides a comprehensive overview of these targeted applications across various specialty oilseed systems, summarizing the specific interaction mechanisms, oilseed sources, and the resulting performance enhancements. This comparative synthesis highlights that the selection of the “optimal assembly pathway” is the primary determinant of whether a complex functions as a high-performance stabilizer or a structural reinforcer.

### 6.1. Fluid-Interfacial and Delivery Systems

Protein–polyphenol complexes serve as high-performance stabilizers in fluid interfaces by forming a multifunctional molecular barrier at the oil–water interface. Unlike native proteins, which typically produce thin and relatively fragile interfacial films, the incorporation of polyphenols—through targeted non-covalent anchoring (e.g., hydrogen bonding and hydrophobic interactions) or controlled covalent grafting—generates a thicker, more viscoelastic, and mechanically robust composite layer [76,119,122]. This enhanced interfacial architecture provides superior resistance to droplet coalescence and oxidative degradation, thereby significantly extending the shelf life of sensitive lipid-based systems. The stabilization operates via a dual-action mechanism: physical steric hindrance and electrostatic repulsion from the protein–polyphenol assembly, combined with chemical radical quenching by the active phenolic hydroxyl groups [89,119].

The practical transition from molecular theory to interfacial performance is demonstrated by the diverse behaviors of specialty oilseed complexes, as synthesized in Table 5. Non-covalent complexes of sunflower protein isolate (SFPI) and chlorogenic acid (CGA) demonstrate the benefits of controlled physical modification. At a neutral pH, hydrogen bonding and hydrophobic interactions between SFPI and CGA significantly reduce oil–water interfacial tension and produce highly stable O/W emulsions resistant to coalescence, while retaining phenolic compounds without the need for complete removal [113].

Building upon this foundational stability, more complex architectures can be achieved through surface-active assembly. In camellia (Camellia oleifera) systems, amphiphilic complexes from Camellia oleifera seed cake protein (COSCP) leverage surface-active assembly to create robust Pickering-type stabilizers. These protein particles anchor at the oil–water interface through hydrophobic pockets and glycosyl-mediated hydrogen bonding, forming stable W/O emulsions (optimal 3% COSCP, oil:water ratio 6.5:3.5) that maintain interfacial integrity and excellent environmental stability for extended periods (up to 28 days) [115].

The highest level of functionalization is observed in the targeted metabolic protection offered by flaxseed protein isolate (FPI). The synergy between flaxseed protein isolate (FPI) and phenolic adducts (FPP/HT) is particularly effective in overcoming the low bioavailability of lipophilic payloads. In FPI-FPP/flaxseed gum nanocomposite coacervates, the interfacial layer protects flaxseed oil from gastric erosion. Consequently, the intestinal release reaches 66–80% (only 5–17% in gastric phase), with markedly modulated lipolysis and improved bioaccessibility of encapsulated bioactives [123].

Despite these functional advantages, translating laboratory prototypes into commercial fluid food systems still requires careful consideration of engineering tolerance. A primary challenge lies in identifying the optimal polyphenol–protein ratio within the formulation window: while moderate phenolic incorporation significantly lowers interfacial tension and enhances emulsion stability [113], excessive loading or alkaline conditions can promote oxidative browning and alter the viscoelastic properties of the interfacial layer. Furthermore, the studies were conducted with purified protein isolates; the inherent structural complexity and competitive macromolecular interactions present in crude oilseed streams may deviate from the well-defined grafting/anchoring kinetics observed in model systems. Future industrial development must therefore focus on establishing precise concentration thresholds and robust processing conditions (pH, shear, temperature) to maximize oxidative protection and long-term emulsion stability while minimizing undesirable sensory changes such as color shifts.

### 6.2. Solid-State Matrices and Active Packaging

In solid-state applications, the interaction between specialty oilseed proteins and polyphenols shifts from interfacial stabilization to the construction of robust three-dimensional networks, as illustrated in Figure 3. Polyphenols serve as multifunctional structural modifiers that bridge polypeptide chains through covalent grafting (via oxidized quinones) and dense non-covalent anchoring (hydrogen bonding and hydrophobic interactions). This molecular reinforcement enables the formation of biodegradable edible films with markedly enhanced barrier properties, inherent antioxidant activity, and potent antimicrobial effects. By modulating phenolic content and cross-linking density, researchers can tailor the optical, mechanical, and bioactive attributes of these materials—transforming fragile protein isolates into active, bio-based packaging systems suitable for food preservation and agricultural mulching applications.

The practical utility of these “naturally activated” matrices is best exemplified by the valorization of sunflower protein streams. Sunflower protein films reinforced with endogenous phenolic compounds (primarily chlorogenic acid) exhibit potent antioxidant activity (ABTS^+^ radical scavenging) while inducing distinct optical changes. As phenolic content increases, the films develop greenish tones with absorption maxima at ~420 nm and ~670 nm (similar to chlorophyll), accompanied by markedly higher opacity. These properties have been strategically leveraged for agricultural mulching films, where increased light-blocking capacity effectively impedes weed growth [124].

Similarly, the incorporation of olive-derived phenolics (e.g., hydroxytyrosol and oleuropein) into biodegradable film and coating matrices yields active packaging systems with potent antioxidant and antimicrobial properties. These olive byproduct-derived compounds inhibit microbial growth and lipid oxidation at the food–package interface, effectively extending the shelf life of sensitive products. By valorizing abundant agro-industrial residues, such solid-state active materials provide a sustainable, circular-economy-driven alternative to synthetic petroleum-based packaging [125].

Optimizing these solid-state systems requires navigating a functional–esthetic equilibrium where material performance must be balanced against consumer acceptance. While phenolic incorporation markedly enhances antioxidant activity and optical barrier properties, it often results in a significant loss of film transparency. Beyond these visual shifts, establishing robust processing windows for scaling from lab-scale casting to industrial production remains a challenge, as phenolic–protein complexes are sensitive to thermal and mechanical stresses. Future development should focus on fine-tuning phenolic content and employing mild processing conditions or synergistic stabilizers to preserve the desirable bioactive and barrier functions while maintaining acceptable visual appeal and cost-effectiveness of the final bio-packaging product.

### 6.3. Structural Texturization and Sensory Modulation

Beyond stabilization and reinforcement, the assembly of protein–polyphenol complexes plays a decisive role in defining the macro-structural and organoleptic properties of plant-based foods. In high-shear environments such as extrusion, these complexes facilitate the alignment of polypeptide chains into anisotropic fibrous hierarchies, enabling the formation of meat analogs with tunable texture. Simultaneously, they provide a chemical scaffold for sensory modulation: by intervening in oxidative pathways and the Maillard reaction, polyphenols shift the volatile profile away from undesirable “green” or “rancid” notes toward enhanced nutty, roasted, and popcorn-like aromas. This dual modulation effectively tailors both the structural integrity and the sensory landscape of specialty oilseed products [126,127].

HME of sunflower meal–pea protein blends produces fibrous meat analogs with tunable hardness, springiness, and gumminess. Extrusion processing creates anisotropic structures that closely mimic the mouthfeel of animal muscle fibers, while simultaneously reducing anti-nutritional factors such as chlorogenic acid. These textural improvements ensure that the resulting products maintain resilience during domestic cooking processes [11,128].

In sesame-based systems, lignans serve as potent modulators of the volatile profile. By regulating the interplay between the Maillard reaction and lipid oxidation, these phenolics shift the aromatic equilibrium away from “green” or “rancid” notes toward enhanced nutty, roasted, and popcorn-like aromas. This dual-action modulation significantly improves the overall sensory profile, addressing one of the primary consumer barriers to plant-based protein adoption [129].

Advancing these applications requires a sophisticated understanding of the sensory-textural interplay, where improvements in fibrous structure must be achieved without compromising palatability. In high-moisture extrusion of sunflower meal–protein blends, the formation of desirable chewiness and springiness is governed primarily by feed moisture content and barrel temperature profiles. However, these same parameters also influence the Maillard reaction and residual phenolic compounds, potentially shifting volatile profiles toward undesirable notes if not precisely controlled. Concurrently, phenolic modulators such as lignans have been shown to effectively intervene in oxidative pathways and the Maillard reaction, enhancing nutty and roasted aromas while suppressing green or rancid off-flavors. Future research should therefore focus on the synergistic optimization of extrusion parameters and endogenous/exogenous phenolic profiles, enabling the decoupling of structural texturization from undesirable sensory outcomes and delivering plant-based meat analogs with both realistic texture and superior flavor.

## 7. Challenges and Future Perspectives

Although this review establishes a coherent “structure–interaction–function” paradigm for specialty oilseed protein–polyphenol systems, several critical challenges continue to constrain both fundamental understanding and industrial translation.

A primary limitation lies in the scarcity of precise, system-specific quantitative boundaries. While qualitative trends (pH, temperature, polyphenol-to-protein ratio) are well documented, exact thresholds that demarcate the moderate interaction regime—such as the critical molar ratio or oxidation extent that separates enhanced emulsification from macroscopic aggregation—are available for only a few herbaceous systems (e.g., sunflower CGA). Without these numbers, industrial process control remains empirical rather than predictive, hindering the precision engineering of functional ingredients [22].

Furthermore, an inherent “Antioxidant-Digestibility Paradox” emerges across applications: polyphenol-mediated stabilization markedly improves oxidative protection and the bioaccessibility of lipophilic bioactives, yet the same interactions frequently reduce proteolytic accessibility and upper-gastrointestinal protein absorption. Reconciling these opposing effects requires multi-objective optimization that current single-factor studies rarely address. Finally, scalability and regulatory hurdles persist. Most laboratory successes rely on purified isolates and controlled alkaline or enzymatic grafting, conditions that are energy-intensive and difficult to replicate at industrial scale without compromising sensory attributes or generating undesirable color changes.

Future research should prioritize the establishment of system-specific quantitative maps of the “Processing Window” through high-throughput experiments, response surface methodology, and machine learning. The integration of in situ characterization techniques and pilot-scale validation in native oilseed matrices will be essential to bridge the remaining gap between laboratory findings and industrial realities. Interdisciplinary efforts combining food chemistry, process engineering, and computational modeling will accelerate the development of dynamic, multi-objective processing strategies, ultimately enabling the high-value valorization of specialty oilseed byproducts in sustainable food systems.

## Figures and Tables

**Table 1:** Integrated analysis of chemical archetypes and predicted binding modes in specialty oilseeds.

Crop System	Dominant Polyphenol	Chemical Archetype	MW (g/mol)	LogP (XLogP3-AA)	Predicted Binding Mode and Residues
Sunflower	Chlorogenic Acid (CGA)	Reactive/Small	354.3	−0.4	Surface-covalent modification; covalent Michael addition to Lys/Cys
Flaxseed	Secoisolariciresinol diglucoside (SDG)	Steric/Glycosylated	686.7	−0.7	Surface anchoring; H-bonding with peptide backbone
Sesame	Sesamol	Reactive/Small	138.1	1.2	Hydrophobic insertion into non-polar pockets; H-bonding
Camellia oleifera	Kaempferol glycosides	Steric/Glycosylated	~448–610	~0.7	Amphiphilic anchoring; interfacial assembly via H-bonding
Walnut	Ellagitannins	Multidentate	>900	0.2	Multidentate surface bridging; H-bonding and aromatic stacking
Olive	Hydroxytyrosol (HT)	Reactive/Small	154.2	−0.7	Oxidative covalent grafting; covalent Michael addition to Lys/Cys

**Table 2:** Structural traits and protein interaction comparisons of polyphenols from specialty oils.

Oilseed System	Dominant Endogenous Polyphenol	Structural Cue Relevant to Binding	Typical Location or Accessibility	Interaction Mechanism	Reference	Evidence Boundary/Key Limitation
Sunflower	Chlorogenic Acid (CGA)	Small, polar o-diphenol that oxidizes readily	Seed and defatted meal; generally accessible during extraction	Often shifts from non-covalent association to quinone-mediated covalent reaction under alkaline or oxidative conditions	[20,57,58,59,60]	Comparatively strong homologous evidence, but the useful window still depends on pH, oxygen, and CGA-to-protein ratio
Flaxseed	Secoisolariciresinol diglucoside (SDG) and lignan-rich phenolic fractions	Bulky, glycosylated, and often matrix-associated	Hull-rich fractions; accessibility depends on dehulling and extraction history	More often surface association or matrix-enabled complexation than rapid oxidation chemistry	[61,62,63,64,65]	Direct site-specific evidence in endogenous systems remains limited
Sesame	Sesamol, sesamin, and related derivatives	Broad polarity continuum from small hydrophobic species to polar glycosides	Distributed across oil-associated and meal-associated fractions	Binding depth and reversibility appear strongly structure-dependent	[30,33,66,67]	Many mechanistic studies rely on model proteins or purified fractions rather than native sesame matrices
Camellia oleifera	Kaempferol glycosides/Catechins	Amphiphilic aromatic sugar architecture.	Meal extracts with interfacial relevance	Dual anchoring through hydrophobic contact plus hydrogen bonding is often proposed	[41,68,69]	Residue-level evidence in endogenous proteins is still sparse
Walnut	Ellagitannins/juglone-related phenolics	Bulky multidentate tannins with strong astringency potential	Pellicle and meal fractions; high local enrichment	Surface bridging, dehydration, aggregation, and strong texture effects are common themes	[47,70,71,72]	Functional outcomes are clear, but quantitative thresholds for beneficial vs. adverse binding remain under-defined
Olive	Hydroxytyrosol (HT)/Oleuropein	Reactive catechol or secoiridoid chemistry	Pomace and meal rather than the oil phase	Oxidation-mediated grafting is often proposed, especially in film systems	[73,74,75]	Evidence is stronger in formulated systems than in fully native olive protein matrices

**Table 3:** Comparative roles, major readouts, and limitations of common characterization methods used for specialty oilseed protein–polyphenol systems.

Method	Main Information Obtained	Strength	Main Limitation	Most Useful for
UV-vis	Chromophore environment and oxidation-related peak shifts	Rapid screening of interaction-induced spectral change	Indirect and weak in structural specificity	Following chromophore perturbation and possible quinone formation
FT-IR/CD	Secondary structure redistribution and folding or unfolding trends	Useful for comparing conformational direction of change	Cannot by itself prove residue-level binding or covalent linkage	Assessing ordered-to-disordered transitions
ITC	Binding constant, stoichiometry, enthalpy, and entropy	Quantitative thermodynamic description in simplified systems	Sensitive to sample purity and less informative in highly heterogeneous matrices	Estimating binding affinity and dominant driving force
Molecular docking	Candidate binding sites and residue contacts	Generates residue-level hypotheses efficiently	Static and strongly dependent on model quality	Proposing plausible binding regions for later validation
Molecular dynamics	Trajectory stability, flexibility, SASA, and compactness	Adds dynamic context to docking-derived models	Computationally model-dependent and still indirect	Testing mechanistic plausibility alongside experiments

**Table 4:** Quantitative landscape of protein–polyphenol interactions and functional thresholds.

Oilseed System	Dominant Interaction	Key Quantitative Parameter	Critical Threshold/Condition	Functional Outcome (Window-dependent)	Reference
Sunflower (SFPI and CGA)	Non-covalent + Covalent	Apparent CGA:protein molar ratio; % bound CGA	Non-covalent: any ratio Covalent: ≥ 1:1 (pH 9)	Improved solubility; max gel strength at 1:1 (covalent); green color + weakened gel at >1:1	[11,113]
Flaxseed (FPI and FPP)	Non-covalent + Covalent	Bound phenolics 20–30%	pH 9.0, 24 h oxidation	Emulsion oxidative improved stability; TBARS levels decreased; solubility and particle size changes	[16,114]
Sesame (Sesamol and Myosin/WPI)	Non-covalent (H-bond + hydrophobic)	Ka = 1.44 × 10^5^ M^−1^ (*n* = 1.33)	0.09% (w/v) sesamol	Emulsion stability increased; oxidative stability increased by 72.6%; particle size decreased	[33,66]
Camellia oleifera (COSCP and EGCG/SLS & FG)	Non-covalent (particle adsorption) + interfacial synergy	Encapsulation efficiency 82.3%	COSCP 3% + oil:water = 6.5:3.5; FG:SLS = 0.167	Bioaccessibility increased by 30.8%; controlled release (26.81% at 4 h); oxidative stability increased	[41,115]
Walnut (WPP and WalPI)	Non-covalent (dominant) + Covalent	Polyphenol content (19.64 mg GAE/g)	pH 9.0 extraction	β-sheet decreased, while random coil, α-helix and β-turn increased (protein unfolding); WHC increased, FC and FS increased; surface hydrophobicity decreased by 62.9%, OHC decreased, and EAI, solubility and thermal stability decreased	[45,80]
Olive (OP/OOP and Gelatin)	Non-covalent + Covalent	Optimal addition 2% w/w	Melt extrusion or laccase; 2% polyphenol	Tensile strength increased by 102.25%; antioxidant activity increased; thermal stability increased	[83]

**Table 5:** Strategic classification and core functional gains of specialty oilseed protein–polyphenol complexes in food systems.

Section	Application Category	Oilseed System	Core Functional Gain
Section 6.1	Fluid-Interfacial and Delivery Systems	Camellia oleifera Sunflower Flaxseed	• Stable gel-like W/O Pickering emulsion with EGCG encapsulation 82.3%, bioaccessibility increased by 30.8%, controlled release • SFPI-CGA complexes significantly reduce interfacial tension and enhance O/W emulsion coalescence stability • FPI-phenolic coacervates enable intestinal-targeted FO release with modulated lipolysis
Section 6.2	Solid-State Matrices and Active Packaging	Sunflower Olive	• Naturally activated biodegradable films with built-in antioxidant activity • Increased opacity + greenish tones for mulching films • Active packaging with oxidative protection and extended shelf life for sensitive foods
Section 6.3	Structural Texturization and Sensory Modulation	Sunflower Sesame	• High-moisture extrusion produces fibrous meat analogs with tunable hardness, springiness, gumminess • Sesame lignans modulate Maillard + lipid oxidation → enhanced nutty/roasted sesame/popcorn aromas, suppressed green/rancid notes • Overall improved sensory profile and texture in plant-based foods

## Data Availability

No new data were created or analyzed in this study. Data sharing is not applicable to this article.

## Abbreviations

The following abbreviations are used in this manuscript:

AEF	Alkaline-extracted fraction
ARG	Arginine
ASN	Asparagine
ASP	Aspartic acid
BSA	Bovine serum albumin
CD	Circular dichroism
CGA	Chlorogenic acid
COSCP	Camellia oleifera seed cake protein
Cys	Cysteine
EAI	Emulsifying activity index
EGCG	Epigallocatechin gallate
FC	Foaming capacity
FG	Flavonoid glycosides from Camellia oleifera cake
FO	Fish oil
FP	Flaxseed polyphenols
FPI	Flaxseed protein isolate
FPP	Flaxseed polyphenols
FRAP	Ferric-reducing antioxidant power
FT-IR	Fourier transform infrared spectroscopy
GLN	Glutamine
GLU	Glutamic acid
His	Histidine
HME	High-moisture extrusion
HT	Hydroxytyrosol
ITC	Isothermal titration calorimetry
Lys	Lysine
MD	Molecular dynamics
MW	Molecular weight
OHC	Oil-holding capacity
O/W	Oil-in-water
OP	The olive polyphenols
OOP	Both OP and oxidized olive polyphenols
ORAC	Oxygen radical absorbance capacity
Rg	Radius of gyration
RMSD	Root-mean-square deviation
RMSF	Root-mean-square fluctuation
RP-HPLC	Reversed-phase high-performance liquid chromatography
RSM	Surface response methodology
SASA	Solvent-accessible surface area
SDG	Secoisolariciresinol diglucoside
SEF	Salt-extracted fraction
SER	Serine
SFPI	Sunflower protein isolate
SLS	Sodium N-lauroylsarcosinate
SPR	Surface plasmon resonance
TBARS	Thiobarbituric acid reactive substances
Tp	Temperature
UV-vis	Ultraviolet-visible spectroscopy
WalPI	Walnut protein isolate
WEF	Water-extracted fraction
WHC	Water-holding capacity
W/O	Water-in-oil
WPI	Whey protein isolate
WPP	Walnut pellicle polyphenols
ZD	Zeaxanthin dipalmitate
β-LG	β-lactoglobulin

## References

[1] Garbacz K., Wawrzykowski J., Czelej M., Czernecki T., Waśko A. (2023). Recent Trends in the Application of Oilseed-Derived Protein Hydrolysates as Functional Foods. Foods.

[2] Kotecka-Majchrzak K., Sumara A., Fornal E., Montowska M. (2020). Oilseed proteins–Properties and application as a food ingredient. Trends Food Sci. Technol..

[3] Mueed A., Shibli S., Korma S.A., Madjirebaye P., Esatbeyoglu T., Deng Z. (2022). Flaxseed Bioactive Compounds: Chemical Composition, Functional Properties, Food Applications and Health Benefits-Related Gut Microbes. Foods.

[4] Mahendra Kumar C., Singh S.A. (2015). Bioactive lignans from sesame (*Sesamum indicum* L.): Evaluation of their antioxidant and antibacterial effects for food applications. J. Food Sci. Technol..

[5] Qiu Y., He D., Yang J., Ma L., Zhu K., Cao Y. (2020). Kaempferol separated from Camellia oleifera meal by high-speed countercurrent chromatography for antibacterial application. Eur. Food Res. Technol..

[6] Medic A., Jakopic J., Hudina M., Solar A., Veberic R. (2021). Identification and quantification of the major phenolic constituents in *Juglans regia* L. peeled kernels and pellicles, using HPLC-MS/MS. Food Chem..

[7] Omar S.H. (2010). Oleuropein in olive and its pharmacological effects. Sci. Pharm..

[8] Wildermuth S.R., Young E.E., Were L.M. (2016). Chlorogenic Acid Oxidation and Its Reaction with Sunflower Proteins to Form Green-Colored Complexes. Compr. Rev. Food Sci. Food Saf..

[9] Zhang K., Huang J., Wang D., Wan X., Wang Y. (2024). Covalent polyphenols-proteins interactions in food processing: Formation mechanisms, quantification methods, bioactive effects, and applications. Front. Nutr..

[10] Rawel H.M., Rohn S. (2010). Nature of hydroxycinnamate-protein interactions. Phytochem. Rev..

[11] Jia W., Sethi D.S., van der Goot A.J., Keppler J.K. (2022). Covalent and non-covalent modification of sunflower protein with chlorogenic acid: Identifying the critical ratios that affect techno-functionality. Food Hydrocoll..

[12] Liang Y., Were L. (2020). Cysteine’s effects on chlorogenic acid quinone induced greening and browning: Mechanism and effect on antioxidant reducing capacity. Food Chem..

[13] Liu F., Sun C., Yang W., Yuan F., Gao Y. (2015). Structural characterization and functional evaluation of lactoferrin-polyphenol conjugates formed by free-radical graft copolymerization. RSC Adv..

[14] Zhang Y., Xiao H., Lv X., Wang D., Chen H., Wei F. (2022). Comprehensive review of composition distribution and advances in profiling of phenolic compounds in oilseeds. Front. Nutr..

[15] Teh S.-S., Bekhit A.E.-D., Birch J. (2014). Antioxidative Polyphenols from Defatted Oilseed Cakes: Effect of Solvents. Antioxidants.

[16] Alu’datt M.H., Rababah T., Ereifej K., Brewer S., Alli I. (2013). Phenolic–protein interactions in oilseed protein isolates. Food Res. Int..

[17] Zhang W. (2020). High quality development in direction and countermeasures of specialized oil industry in China. Chin. J. Oil Crop Sci..

[18] Zhou W., He J. (2025). Local Agency in Crop Booms and Busts: Insights from the Walnut Plantations of Southwest China. Mt. Res. Dev..

[19] Sun J., Huang D., Xia S., Zhang Y., Tao J. (2024). Research progress of woody oil crops in China: A review. Seed Biol..

[20] Gomes A., Cangussu L.B., Cunha R.L., Oliveira L.S.d., Franca A.S., Costa A.L.R. (2025). Investigating the Impact of Chlorogenic Acid Content and Cellulose Nanoparticles on Sunflower Protein-Based Emulsions and Films. Foods.

[21] Wang L., Pan X., Jiang L., Chu Y., Gao S., Jiang X., Zhang Y., Chen Y., Luo S., Peng C. (2022). The Biological Activity Mechanism of Chlorogenic Acid and Its Applications in Food Industry: A Review. Front. Nutr..

[22] Tarahi M., Gharagozlou M., Niakousari M., Hedayati S. (2024). Protein–Chlorogenic Acid Interactions: Mechanisms, Characteristics, and Potential Food Applications. Antioxidants.

[23] Ha S.M.L., Schild K., Heyn T.R., Marel A.-K., Schwarz K., de Bruijn W.J.C., Keppler J.K. (2026). Covalent protein-phenolic modification–Effect of the phenolic compound structure on protein modification and conformational changes. Food Hydrocoll..

[24] Kezimana P., Dmitriev A.A., Kudryavtseva A.V., Romanova E.V., Melnikova N.V. (2018). Secoisolariciresinol Diglucoside of Flaxseed and Its Metabolites: Biosynthesis and Potential for Nutraceuticals. Front. Genet..

[25] Fuentealba C., Figuerola F., Estevez A.M., González-Muñoz A., Muñoz O. (2015). Optimization of secoisolariciresinol diglucoside extraction from flaxseed (*Linum usitatissimum* L.) and isolation by a simple HPLC-UV method. CyTA J. Food.

[26] Cheng C., Wang L., Yu X., Huang F., Yang J., Geng F., Xia X., Xiang X., Xu S., Deng Q. (2024). Structural identification and antioxidative activity evaluation of flaxseed lignan macromolecules: Structure-activity correlation. Food Sci. Hum. Wellness.

[27] Plaha N.S., Awasthi S., Sharma A., Kaushik N. (2022). Distribution, biosynthesis and therapeutic potential of lignans. 3 Biotech.

[28] Ford J.D., Huang K.S., Wang H.B., Davin L.B., Lewis N.G. (2001). Biosynthetic pathway to the cancer chemopreventive secoisolariciresinol diglucoside-hydroxymethyl glutaryl ester-linked lignan oligomers in flax (*Linum usitatissimum*) seed. J. Nat. Prod..

[29] Adolphe J.L., Whiting S.J., Juurlink B.H., Thorpe L.U., Alcorn J. (2010). Health effects with consumption of the flax lignan secoisolariciresinol diglucoside. Br. J. Nutr..

[30] Mostashari P., Mousavi Khaneghah A. (2024). Sesame Seeds: A Nutrient-Rich Superfood. Foods.

[31] Lee S.W., Jeung M.K., Park M.H., Lee S.Y., Lee J. (2010). Effects of roasting conditions of sesame seeds on the oxidative stability of pressed oil during thermal oxidation. Food Chem..

[32] Arab R., Casal S., Pinho T., Cruz R., Freidja M.L., Lorenzo J.M., Hano C., Madani K., Boulekbache-Makhlouf L. (2022). Effects of Seed Roasting Temperature on Sesame Oil Fatty Acid Composition, Lignan, Sterol and Tocopherol Contents, Oxidative Stability and Antioxidant Potential for Food Applications. Molecules.

[33] Gao Z., Ji Z., Wang L., Deng Q., Quek S.Y., Liu L., Dong X. (2023). Improvement of Oxidative Stability of Fish Oil-in-Water Emulsions through Partitioning of Sesamol at the Interface. Foods.

[34] Andargie M., Vinas M., Rathgeb A., Möller E., Karlovsky P. (2021). Lignans of Sesame (*Sesamum indicum* L.): A Comprehensive Review. Molecules.

[35] Fhaner M., Hwang H.-S., Winkler-Moser J., Bakota E., Liu S. (2015). Protection of fish oil from oxidation with sesamol. Eur. J. Lipid Sci. Technol..

[36] Geetha T., Rohit B., Pal K.I. (2009). Sesamol: An efficient antioxidant with potential therapeutic benefits. Med. Chem..

[37] Dossou S.S.K., Xu F.-t., Dossa K., Zhou R., Zhao Y.-z., Wang L.-h. (2023). Antioxidant lignans sesamin and sesamolin in sesame (*Sesamum indicum* L.): A comprehensive review and future prospects. J. Integr. Agric..

[38] Xie Y., Wang Y., Xie J., Yu Q., Lu H., Zhong J., Chen Y. (2023). Camellia oleifera seeds cake: Polyphenol profile and in vitro antioxidant activities as determined by different harvest periods. Food Biosci..

[39] Cheng G., Zhu J., Si J., Wu T., Chen J., Xu X., Feng S., Chen T., Ding C., Zhou L. (2024). Optimization of ultrasound-assisted enzymatic extraction, chemical constituents, biological activities, and stability of Camellia oleifera fruit shell brown pigments. LWT.

[40] Zhang D., Nie S., Xie M., Hu J. (2020). Antioxidant and antibacterial capabilities of phenolic compounds and organic acids from Camellia oleifera cake. Food Sci. Biotechnol..

[41] Li W., Zhang F., Han C., Li P., Jiang J., Zhu L. (2024). Synergy evaluation of flavonoid glycosides from Camellia oleifera cake and ionic surfactants on the stability of oil-in-water emulsions. J. Clean. Prod..

[42] Yatheshappa G.K., Farooq S., Zhang H. (2025). Effects of polar quercetin and non-polar resveratrol on the functional properties, oxidative stability and interfacial behavior of camellia oil body emulsions. Food Chem..

[43] Luo S.-Z., Hu X.-F., Pan L.-H., Zheng Z., Zhao Y.-Y., Cao L.-L., Pang M., Hou Z.-G., Jiang S.-T. (2019). Preparation of camellia oil-based W/O emulsions stabilized by tea polyphenol palmitate: Structuring camellia oil as a potential solid fat replacer. Food Chem..

[44] Fukuda T., Ito H., Yoshida T. (2003). Antioxidative polyphenols from walnuts (*Juglans regia* L.). Phytochemistry.

[45] Zhan Y., Ma M., Chen Z., Ma A., Li S., Xia J., Jia Y. (2023). A Review on Extracts, Chemical Composition and Product Development of Walnut *Diaphragma Juglandis* Fructus. Foods.

[46] Shimoda H., Tanaka J., Kikuchi M., Fukuda T., Ito H., Hatano T., Yoshida T. (2009). Effect of polyphenol-rich extract from walnut on diet-induced hypertriglyceridemia in mice via enhancement of fatty acid oxidation in the liver. J. Agric. Food Chem..

[47] Kong X., Huang Z., Zhang C., Hua Y., Chen Y., Li X. (2023). Phenolic compounds in walnut pellicle improve walnut (*Juglans regia* L.) protein solubility under pH-shifting condition. Food Res. Int..

[48] Mateș L., Banc R., Zaharie F.A., Rusu M.E., Popa D.-S. (2024). Mechanistic Insights into the Biological Effects and Antioxidant Activity of Walnut (*Juglans regia* L.) Ellagitannins: A Systematic Review. Antioxidants.

[49] Romeu M.F.C., Bernardo J., Daniel C.I., Costa N., Crespo J.G., Silva Pinto L., Nunes da Ponte M., Nunes A.V.M. (2024). Hydroxytyrosol recovery from olive pomace: A simple process using olive mill industrial equipment and membrane technology. J. Food Sci. Technol..

[50] Wong Y.L., Boulos S., Nyström L. (2025). The olive biophenol hydroxytyrosol in neutral aqueous solutions—A UPLC-MS/MS investigation of its stability and oxidative color formation. Front. Nutr..

[51] Cravotto C., Fabiano-Tixier A.S., Claux O., Rapinel V., Tomao V., Stathopoulos P., Skaltsounis A.L., Tabasso S., Jacques L., Chemat F. (2022). Higher Yield and Polyphenol Content in Olive Pomace Extracts Using 2-Methyloxolane as Bio-Based Solvent. Foods.

[52] Gao Y., Liu W., Pan S., Li J., Wang J., Chen L., Ma X., Leng H. (2025). Hydroxytyrosol: Biological activities and potential application in livestock production. Front. Vet. Sci..

[53] Akazawa T., Itami H., Furumoto T., Nozaki C., Koike H., Iritani S., Amimoto N., Ogawa M. (2021). Impact of an Olive Leaf Polyphenol 3,4-DHPEA-EDA on Physical Properties of Food Protein Gels. J. Agric. Food Chem..

[54] Luzi F., Pannucci E., Clemente M., Grande E., Urciuoli S., Romani A., Torre L., Puglia D., Bernini R., Santi L. (2021). Hydroxytyrosol and Oleuropein-Enriched Extracts Obtained from Olive Oil Wastes and By-Products as Active Antioxidant Ingredients for Poly (Vinyl Alcohol)-Based Films. Molecules.

[55] Ozdal T., Capanoglu E., Altay F. (2013). A review on protein–phenolic interactions and associated changes. Food Res. Int..

[56] Meng Y., Li C. (2021). Conformational changes and functional properties of whey protein isolate-polyphenol complexes formed by non-covalent interaction. Food Chem..

[57] Prigent S.V., Voragen A.G., Visser A.J., van Koningsveld G.A., Gruppen H. (2007). Covalent interactions between proteins and oxidation products of caffeoylquinic acid (chlorogenic acid). J. Sci. Food Agric..

[58] Sabir M.A., Sosulski F.W., Finlayson A.J. (1974). Chlorogenic acid-protein interactions in sunflower. J. Agric. Food Chem..

[59] Karefyllakis D., Salakou S., Bitter J.H., van der Goot A.J., Nikiforidis C.V. (2018). Covalent Bonding of Chlorogenic Acid Induces Structural Modifications on Sunflower Proteins. ChemPhysChem.

[60] Ishii A.K., Toto Pacioles C., Were L. (2021). Color and structural modifications of alkaline extracted sunflower protein concentrates and isolates using L-cysteine and glutathione. Food Res. Int..

[61] Guimarães Drummond e Silva F., Miralles B., Hernández-Ledesma B., Amigo L., Iglesias A.H., Reyes Reyes F.G., Netto F.M. (2017). Influence of Protein–Phenolic Complex on the Antioxidant Capacity of Flaxseed (*Linum usitatissimum* L.) Products. J. Agric. Food Chem..

[62] Pham L.B., Wang B., Zisu B., Adhikari B. (2019). Covalent modification of flaxseed protein isolate by phenolic compounds and the structure and functional properties of the adducts. Food Chem..

[63] Cheng C., Yu X., McClements D.J., Huang Q., Tang H., Yu K., Xiang X., Chen P., Wang X., Deng Q. (2019). Effect of flaxseed polyphenols on physical stability and oxidative stability of flaxseed oil-in-water nanoemulsions. Food Chem..

[64] Nie C., Qin X., Duan Z., Huang S., Yu X., Deng Q., Xiang Q., Geng F. (2022). Comparative structural and techno-functional elucidation of full-fat and defatted flaxseed extracts: Implication of atmospheric pressure plasma jet. J. Sci. Food Agric..

[65] Cheng C., Yu X., Geng F., Wang L., Yang J., Huang F., Deng Q. (2022). Review on the Regulation of Plant Polyphenols on the Stability of Polyunsaturated-Fatty-Acid-Enriched Emulsions: Partitioning Kinetic and Interfacial Engineering. J. Agric. Food Chem..

[66] Han P., An N., Yang L., Ren X., Lu S., Ji H., Wang Q., Dong J. (2022). Molecular dynamics simulation of the interactions between sesamol and myosin combined with spectroscopy and molecular docking studies. Food Hydrocoll..

[67] Feng X., Li S., Tang S., Wu W. (2024). Insight into the effect of sesamol on the structural and gel properties of yak myofibrillar proteins. Int. J. Biol. Macromol..

[68] Yatheshappa G.K., Farooq S., Jiang Q., Chen M., Zhang H. (2025). Investigating the effects of polar and non-polar polyphenols on the physicochemical properties and functional characteristics of camellia oil body emulsions. Food Chem..

[69] Zheng L., Chen L., Li J., Liang L., Fan Y., Qiu L., Deng Z. (2019). Two Kaempferol Glycosides Separated from Camellia Oleifera Meal by High-Speed Countercurrent Chromatography and Their Possible Application for Antioxidation. J. Food Sci..

[70] Soares S., Brandão E., García-Estevez I., Fonseca F., Guerreiro C., Ferreira-da-Silva F., Mateus N., Deffieux D., Quideau S., de Freitas V. (2019). Interaction between Ellagitannins and Salivary Proline-Rich Proteins. J. Agric. Food Chem..

[71] Yan C., Zhou Z. (2021). Walnut pellicle phenolics greatly influence the extraction and structural properties of walnut protein isolates. Food Res. Int..

[72] Wang R., Tian X., Li Q., Liao L., Wu S., Tang F., Shen D., Liu Y. (2022). Walnut pellicle color affects its phenolic composition: Free, esterified and bound phenolic compounds in various colored-pellicle walnuts. J. Food Compos. Anal..

[73] Vilaplana-Pérez C., Auñón D., García-Flores L.A., Gil-Izquierdo A. (2014). Hydroxytyrosol and Potential Uses in Cardiovascular Diseases, Cancer, and AIDS. Front. Nutr..

[74] Rietjens S.J., Bast A., Haenen G. (2007). New insights into controversies on the antioxidant potential of the olive oil antioxidant hydroxytyrosol. J. Agric. Food Chem..

[75] Soleimanifar M., Jafari S.M., Assadpour E. (2020). Encapsulation of olive leaf phenolics within electrosprayed whey protein nanoparticles; production and characterization. Food Hydrocoll..

[76] Li M., Ritzoulis C., Du Q., Liu Y., Ding Y., Liu W., Liu J. (2021). Recent Progress on Protein-Polyphenol Complexes: Effect on Stability and Nutrients Delivery of Oil-in-Water Emulsion System. Front. Nutr..

[77] Li J., Tian R., Liang G., Shi R., Hu J., Jiang Z. (2021). Interaction mechanism of flavonoids with whey protein isolate: A spectrofluorometric and theoretical investigation. Food Chem..

[78] Bordenave N., Hamaker B.R., Ferruzzi M.G. (2014). Nature and consequences of non-covalent interactions between flavonoids and macronutrients in foods. Food Funct..

[79] Huang X., Yan C., Lin M., He C., Xu Y., Huang Y., Zhou Z. (2022). The effects of conjugation of walnut protein isolate with polyphenols on protein solubility, antioxidant activity, and emulsifying properties. Food Res. Int..

[80] Cao Y., Deng Y., Yuan G., Zhao S., Ma Q., Yang J., Guo L., Fan F. (2026). Effects of endogenous walnut pellicle polyphenols on the structure and functional properties of walnut meal isolate proteins. Food Chem. X.

[81] Le Bourvellec C., Renard C.M. (2012). Interactions between polyphenols and macromolecules: Quantification methods and mechanisms. Crit. Rev. Food Sci. Nutr..

[82] Riebel M., Sabel A., Claus H., Fronk P., Xia N., Li H., König H., Decker H. (2015). Influence of Laccase and Tyrosinase on the Antioxidant Capacity of Selected Phenolic Compounds on Human Cell Lines. Molecules.

[83] Wu K., Li Y., Wang H., Xiao J., Ma W., Li L. (2025). High-strength and antioxidant gelatin/(oxidized) olive polyphenol films by melt extrusion method. Food Hydrocoll..

[84] Chen L., Xie X., Li Y., Xiong H., Li L. (2022). Activation mechanism of whey protein isolate mediated by free radicals generated in the ascorbic acid/hydrogen peroxide system. Food Chem..

[85] Wang B., Pham L.B., Adhikari B. (2024). Complexation and conjugation between phenolic compounds and proteins: Mechanisms, characterisation and applications as novel encapsulants. Sustain. Food Technol..

[86] Bayati M., Poojary M.M. (2025). Polyphenol autoxidation and prooxidative activity induce protein oxidation and protein-polyphenol adduct formation in model systems. Food Chem..

[87] Aktaş H., Szpicer A., Strojny-Cieślak B., Borucki W., Schweiggert-Weisz U., Kurek M.A. (2025). Molecular Interplay Between Plant Proteins and Polyphenols: pH as a Switch for Structural and Functional Assembly. Foods.

[88] Huynh H.D., Thi T.H.T., Thi T.X.T., Nargotra P., Wang H.-M.D., Liu Y.-C., Kuo C.-H. (2026). Recent Insights into Protein-Polyphenol Complexes: Molecular Mechanisms, Processing Technologies, Synergistic Bioactivities, and Food Applications. Molecules.

[89] Feng Y., Jin C., Lv S., Zhang H., Ren F., Wang J. (2023). Molecular Mechanisms and Applications of Polyphenol-Protein Complexes with Antioxidant Properties: A Review. Antioxidants.

[90] Sęczyk Ł., Świeca M., Kapusta I., Gawlik-Dziki U. (2019). Protein–Phenolic Interactions as a Factor Affecting the Physicochemical Properties of White Bean Proteins. Molecules.

[91] Eze F.N., Muangrat R., Singh S., Jirarattanarangsri W., Siriwoharn T., Chalermchat Y. (2024). Upcycling of Defatted Sesame Seed Meal via Protein Amyloid-Based Nanostructures: Preparation, Characterization, and Functional and Antioxidant Attributes. Foods.

[92] Schild K., Sönnichsen F.D., Martin D., Garamus V.M., Van der Goot A.J., Schwarz K., Keppler J.K. (2023). Unraveling the effects of low protein-phenol binding affinity on the structural properties of beta-lactoglobulin. Food Chem..

[93] Poklar Ulrih N. (2017). Analytical techniques for the study of polyphenol–protein interactions. Crit. Rev. Food Sci. Nutr..

[94] Karonen M. (2025). Polyphenol–Macromolecule Interactions by Isothermal Titration Calorimetry. Macromol.

[95] Reinmuth-Selzle K., Tchipilov T., Backes A.T., Tscheuschner G., Tang K., Ziegler K., Lucas K., Pöschl U., Fröhlich-Nowoisky J., Weller M.G. (2022). Determination of the protein content of complex samples by aromatic amino acid analysis, liquid chromatography-UV absorbance, and colorimetry. Anal. Bioanal. Chem..

[96] Yan S., Wang Q., Yu J., Li Y., Qi B. (2023). Ultrasound-assisted preparation of protein–polyphenol conjugates and their structural and functional characteristics. Ultrason. Sonochem..

[97] De Meutter J., Goormaghtigh E. (2021). Evaluation of protein secondary structure from FTIR spectra improved after partial deuteration. Eur. Biophys. J..

[98] Usoltsev D., Sitnikova V., Kajava A., Uspenskaya M. (2019). Systematic FTIR Spectroscopy Study of the Secondary Structure Changes in Human Serum Albumin under Various Denaturation Conditions. Biomolecules.

[99] Waszkowiak K., Mikołajczak B., Polanowska K., Wieruszewski M., Siejak P., Smułek W., Jarzębski M. (2023). Protein Fractions from Flaxseed: The Effect of Subsequent Extractions on Composition and Antioxidant Capacity. Antioxidants.

[100] Yang F., Li J., Wang J., Gan S., Dong G. (2026). Covalent modification of flaxseed protein isolate by polyphenols and characterization of the structural and functional properties of the conjugates. Food Chem. X.

[101] Wei Y., Thyparambil A.A., Latour R.A. (2014). Protein helical structure determination using CD spectroscopy for solutions with strong background absorbance from 190 to 230nm. Biochim. Biophys. Acta.

[102] Ma J.-H., Ren L.-Q., Tang T.-X., Chen Y.-Y., Zhang C.-X., Ke Y.-F., Zhang Y., Muskat M.N., Cheng X.-R. (2024). Effects of polyphenols from walnut pellicle on the structure and allergenicity of walnut globulin. Food Biosci..

[103] Claasen B., Xiong M., Mayer P.S., Sogl G., Buchweitz M. (2025). Applying Isothermal Titration Calorimetry and Saturation Transfer Difference-NMR to Study the Mode of Interaction of Flavan-3-ols with α-Amylase to Understand Their Impact on Starch Hydrolysis. J. Agric. Food Chem..

[104] Saponaro A. (2018). Isothermal Titration Calorimetry: A Biophysical Method to Characterize the Interaction between Label-free Biomolecules in Solution. Bio-Protocol.

[105] Shahidi F., Dissanayaka C. (2023). Phenolic-protein interactions: Insight from in-silico analyses—A review. Food Prod. Process. Nutr..

[106] Baruah I., Kashyap C., Guha A.K., Borgohain G. (2022). Insights into the Interaction between Polyphenols and β-Lactoglobulin through Molecular Docking, MD Simulation, and QM/MM Approaches. ACS Omega.

[107] Liu J., Song G., Zhou L., Yuan Y., Wang D., Yuan T., Li L., Yuan H., Xiao G., Gong J. (2023). Recent advances in the effect of ultrasound on the binding of protein−polyphenol complexes in foodstuff. Food Front..

[108] Brudzynski K., Maldonado-Alvarez L. (2015). Polyphenol-Protein Complexes and Their Consequences for the Redox Activity, Structure and Function of Honey. A Current View and New Hypothesis—A Review. Pol. J. Food Nutr. Sci..

[109] Can Karaca A., Tan C., Assadpour E., Jafari S.M. (2025). Recent advances in the plant protein-polyphenol interactions for the stabilization of emulsions. Adv. Colloid Interface Sci..

[110] Ebrahimi P., Lante A., Grossmann L. (2025). Protein-polyphenol complexation vs. conjugation: A review on mechanisms, functional differences, and antioxidant-emulsifier roles. Food Hydrocoll..

[111] Wang Y., Cao S., Meng Y., Cheng Y., Han Z., Wang F. (2024). Mechanisms underlying the effect of walnut pellicle extracts and its four representative polyphenols on in vitro digestion of walnut protein isolate. Food Bioprod. Process..

[112] Yang L., Tang X., Bu N., Liu D. (2025). Encapsulation of zeaxanthin dipalmitate emulsion with flaxseed polyphenol and protein isolate improves its stability and bioaccessibility. Food Res. Int..

[113] Karefyllakis D., Altunkaya S., Berton-Carabin C.C., van der Goot A.J., Nikiforidis C.V. (2017). Physical bonding between sunflower proteins and phenols: Impact on interfacial properties. Food Hydrocoll..

[114] Pham L.B., Wang B., Zisu B., Adhikari B. (2019). Complexation between flaxseed protein isolate and phenolic compounds: Effects on interfacial, emulsifying and antioxidant properties of emulsions. Food Hydrocoll..

[115] Cui C., Wei Z., Hong Z., Zong J., Li H., Peng C., Cai H., Hou R. (2023). Preparation of water-in-oil Pickering emulsion stabilized by Camellia oleifera seed cake protein and its application as EGCG delivery system. LWT.

[116] Das N., Khan T., Halder B., Ghosh S., Sen P. (2024). Macromolecular crowding effects on protein dynamics. Int. J. Biol. Macromol..

[117] Xue H., Feng J., Tang Y., Wang X., Tang J., Cai X., Zhong H. (2024). Research progress on the interaction of the polyphenol–protein–polysaccharide ternary systems. Chem. Biol. Technol. Agric..

[118] Jiang J., Qian S., Song T., Lu X., Zhan D., Zhang H., Liu J. (2024). Food-packaging applications and mechanism of polysaccharides and polyphenols in multicomponent protein complex system: A review. Int. J. Biol. Macromol..

[119] Tang S., Yang X., Wang C., Wang C. (2025). Effects of Polyphenols on the Structure, Interfacial Properties, and Emulsion Stability of Pea Protein: Different Polyphenol Structures and Concentrations. Molecules.

[120] Shahidi F., Athiyappan K.D. (2025). Polyphenol-polysaccharide interactions: Molecular mechanisms and potential applications in food systems—A comprehensive review. Food Prod. Process. Nutr..

[121] Lešnik S., Jukić M., Bren U. (2025). Unveiling polyphenol-protein interactions: A comprehensive computational analysis. J. Cheminform..

[122] Huang A., Luo S., Ning F., Ye J., Liu C. (2024). Preparation of protein-polyphenol-polysaccharide ternary complexes to regulate the interfacial structure of emulsions: Interfacial behavior and emulsion stability. Food Hydrocoll..

[123] Pham L.B., Wang B., Zisu B., Truong T., Adhikari B. (2021). In-vitro digestion of flaxseed oil encapsulated in phenolic compound adducted flaxseed protein isolate-flaxseed gum complex coacervates. Food Hydrocoll..

[124] Salgado P.R., Molina Ortiz S.E., Petruccelli S., Mauri A.N. (2010). Biodegradable sunflower protein films naturally activated with antioxidant compounds. Food Hydrocoll..

[125] Khwaldia K., Attour N., Matthes J., Beck L., Schmid M. (2022). Olive byproducts and their bioactive compounds as a valuable source for food packaging applications. Compr. Rev. Food Sci. Food Saf..

[126] Wang Y., Tuccillo F., Lampi A.-M., Knaapila A., Pulkkinen M., Kariluoto S., Coda R., Edelmann M., Jouppila K., Sandell M. (2022). Flavor challenges in extruded plant-based meat alternatives: A review. Compr. Rev. Food Sci. Food Saf..

[127] Wang H., Luo S., Hu X., Liu C. (2025). Gallic acid enhanced fiber formation in soy protein isolate-based meat analogues. Food Chem..

[128] Cai M., Kadam A., House J.D., Koksel F. (2026). High-moisture extrusion modifies texture and improves nutritional value of sunflower meal-pea protein meat analogues. Appl. Food Res..

[129] Yin W.-t., Yang C.-j., Yang H.-j., Hu B.-b., Zhang F., Wang X.-d., Liu H.-m., Miao H.-m. (2024). Sesame lignans modulate aroma formation in sesame oil through the Maillard reaction and lipid oxidation in model systems. Food Chem..

